# Dr. William T. Griffin

**Published:** 1909-09

**Authors:** 


Dr. William T. Griffin, of Buffalo, died at the emergency Hospital, June 5, 1909, aged 32 years. His death was reported as due to cerebral hemorrhage as determined by an autopsy. He graduated from Niagara University in 1898.

